# Adenoviral Vector-Based Vaccine Platform for COVID-19: Current Status

**DOI:** 10.3390/vaccines11020432

**Published:** 2023-02-13

**Authors:** Vivek P. Chavda, Rajashri Bezbaruah, Disha Valu, Bindra Patel, Anup Kumar, Sanjay Prasad, Bibhuti Bhusan Kakoti, Ajeet Kaushik, Mariya Jesawadawala

**Affiliations:** 1 Department of Pharmaceutics and Pharmaceutical Technology, L. M. College of Pharmacy, Ahmedabad 380009, Gujarat, India; 2 Department of Pharmaceutical Sciences, Faculty of Science and Engineering, Dibrugarh University, Dibrugarh 786004, Assam, India; 3 Drug Product Development Laboratory, Biopharma Division, Intas Pharmaceutical Ltd., Moraiya, Ahmedabad 382213, Gujarat, India; 4 Pharmacy Section, L. M. College of Pharmacy, Ahmedabad 380009, Gujarat, India; 5 Cell and Gene Therapy Drug Product Development Laboratory, Biopharma Division, Intas Pharmaceutical Ltd., Moraiya, Ahmedabad 382213, Gujarat, India; 6 NanoBioTech Laboratory, Health Systems Engineering, Department of Environmental Engineering, Florida Polytechnic University, Lakeland, FL 33805-8531, USA

**Keywords:** viral vector, SARS-CoV-2, variants, Ad Vector, nasal vaccine, intramuscular vaccine, intranasal vaccine, COVID

## Abstract

The coronavirus disease (COVID-19) breakout had an unimaginable worldwide effect in the 21st century, claiming millions of lives and putting a huge burden on the global economy. The potential developments in vaccine technologies following the determination of the genetic sequence of SARS-CoV-2 and the increasing global efforts to bring potential vaccines and therapeutics into the market for emergency use have provided a small bright spot to this tragic event. Several intriguing vaccine candidates have been developed using recombinant technology, genetic engineering, and other vaccine development technologies. In the last decade, a vast amount of the vaccine development process has diversified towards the usage of viral vector-based vaccines. The immune response elicited by such vaccines is comparatively higher than other approved vaccine candidates that require a booster dose to provide sufficient immune protection. The non-replicating adenoviral vectors are promising vaccine carriers for infectious diseases due to better yield, cGMP-friendly manufacturing processes, safety, better efficacy, manageable shipping, and storage procedures. As of April 2022, the WHO has approved a total of 10 vaccines around the world for COVID-19 (33 vaccines approved by at least one country), among which three candidates are adenoviral vector-based vaccines. This review sheds light on the developmental summary of all the adenoviral vector-based vaccines that are under emergency use authorization (EUA) or in the different stages of development for COVID-19 management.

## 1. Introduction

**Figure 1:**
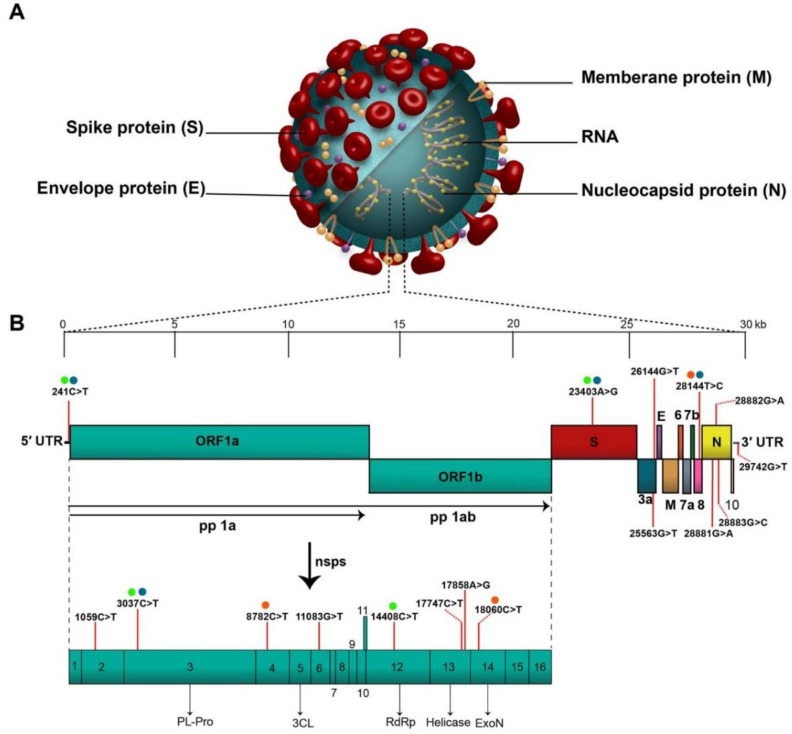
**Novel coronavirus structure and genomic backbone.** “(**A**). Structure of SARS-CoV-2 and (**B**). The SARS-CoV-2 genome (29,903 nucleotides) comprises the 5′ UTR, ORF1a/b encoding 16 nsps for replication, four genes that encode structural proteins including S, E, M, and N proteins, six accessory genes that encode six accessory proteins such as ORF3a, ORF6, ORF7a, ORF7b, ORF8, and ORF10, as well as the 3′ UTR. The location of the seventeen high-frequency mutations and co-mutations reported in the literature are shown on the genome by vertical red lines and circles with similar color, respectively. Abbreviations: SARS-CoV-2, severe acute respiratory syndrome coronavirus 2; 5′ UTR, 5′ untranslated region; OFR, open reading frame; nsp, non-structural protein.” (Reprint with permission from Elsevier).

The coronavirus disease (COVID-19), which was discovered for the first time in Wuhan, China, in December 2019, was declared a pandemic by the WHO on March 11, 2020 [1]. The coronavirus known as “severe acute respiratory syndrome coronavirus-2 (SARS-CoV-2)” is the cause of this infection. The virus was initially identified as 2019-nCoV [2,3], but later became known as SARS-CoV-2 because it shared genetic similarities with the coronavirus infection that caused the SARS outbreak in 2003 [4,5]. The genomic backbone of the SARS-CoV-2 virus is highlighted in Figure 1; to know more about the genomic organization of SARS-CoV-2, readers are recommended to refer to the work of Rahimi and co-workers [6]. More than 42,000 SARS-CoV-2 genomic sequences have been reported so far in “Global Initiative on Sharing All influenza Data (GISAID)” [7]. To fight coronavirus disease initially, the treatments included broad spectrum antibiotics and specific antiviral and NSAIDs (non-steroidal anti-inflammatory drugs) with immunotherapy and adjunctive therapy [8]. In this context, use of interleukin (IL-6, IL-1) and hyper-immune immunoglobulin inhibitors, chloroquine or hydroxychloroquine, remdesivir, corticosteroids, mechanical ventilation, immunotherapy and stem cell therapy are under clinical implications in COVID-19 patients [1,9,10,11].

Following the announcement of the SARS-CoV-2 genetic sequence, intensive worldwide R&D activities were performed to create a vaccine to curtail the pandemic [12,13,14]. Several vaccine candidates with promising potential have been developed using recombinant technologies, genetic engineering, and other vaccine development technologies. According to data published by WHO until December 2022, 175 vaccines are in the clinical development stage while 199 vaccines are in the preclinical phase, with 13 vaccines candidates approved for EUL [15]. The world is just recovering from the devastating effects of the delta and delta plus variants, and a new variant has surfaced named omicron [16,17,18,19]. The omicron variant genetic variations (around 50 mutations) are known to increase risk of transmission, higher viral binding affinity, and immune response escape [20]. A combined mitigation approach of immunization and public health policies is anticipated to remain an efficient approach.

Vaccines usually uses natural or synthetic viral antigenic material to induce immunity in the host against the virus, however, the procedure remains different with diverse vaccine platforms. On the other hand, vaccines based on viral vectors use a viral vector that, when injected, activates the machinery of the host cell to create the viral antigen [21,22]. The vaccine mimics the pathogenesis of a naturally occurring infection with the same pathogens, mainly viruses, by infecting cells and forcing them to generate enormous quantities of antigen when an immune response is triggered [23]. The rVSV-ZEBOV vaccine is an illustration of a viral vector vaccine [24]. Imojev, a vaccine against Japanese Encephalitis, was the first viral vector-based vaccine authorized for clinical application in 2011 [25]. A wide range of viruses viz. poxviruses, lentivirus, adenoviruses, paroviruses, adeno-associated viruses, measles viruses, togaviruses etc. have been utilized as a source for establishing viral vector-based vaccines [26].

Viruses are used as replicating (typically attenuated) or non-replicating vectors in this vaccine platform [22,27]. Replicating viral vector-based vaccines invade cells, which subsequently produces vaccination antigens and new viruses which afterwards may contaminate another cell and manifest immunogenicity. Non-replicating viral vector vaccines, on the other hand, can create vaccine antigens but not new virus particles after entering the cell [28]. In the journey of making effective vaccine at the fast speed against various infectious disease, adenoviral vectors have been always favored, and the same is the case with COVID-19. As of 1 November 2022 there are 27 viral vector vaccines under development, most of which (almost 20) are adenovirus-based vaccines (10 in phase III, 5 in phase II and 9 in phase I) [15]. Up until December, 2022, four viral vector vaccines viz. Ad26.COV2.S, Vaxzevria, Covishield (Oxford/AstraZeneca formulation) and Convidecia have been included in the Emergency Use Listing (EUL) by WHO [29].

## 2. Adenoviruses (AdVs) as a Vector for Vaccine Delivery

**Figure 2:**
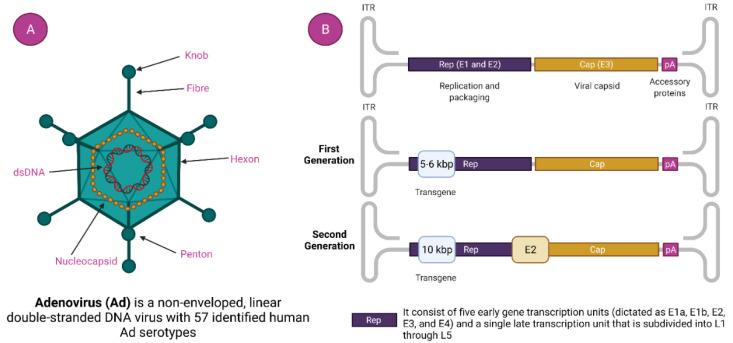
**Adenovirus and adenovirus-based vector.** (**A**). Adenovirus Structure and (**B**). Generation of Adenoviral Vectors.

Adenovirus has gained considerable consideration as an important therapeutic vector, and it was the initial DNA virus to undergo rigorous medical intervention, owing to its very suitable biology, stable genes, better transduction efficiency, and ease of mass production [30,31]. Adenoviruses are linear double-stranded DNA viruses with the genomic size of 34 to 43 kb and without any envelope covering. They are typically to blame for minor, ocular and respiratory infections in people [30,32]. Its nucleocapsid consists of many proteins with three major types: fiber, penton, and hexon (Figure 2). More than 150 primate AdVs have been identified, and numerous AdVs are being developed for vaccination purposes. Adenoviruses, unlike other viruses, do not attach to the host genome; instead, they remain in an episomal condition [33]. Adenovirus can be isolated from varieties of human to various animal species such as chimpanzee [34]. About 50 serotypes types of adenovirus have been isolated in humans, which are further sub-grouped into seven subtypes (A–G) depending on their homology sequence and capacity to form erythrocyte agglomerates [35]. AdVs are more affordable and thermostable than mRNA vaccines. Furthermore, their adaptable viral biology allows for the expansion of vectors with better vaccination efficiency, allowing for a thorough assessment of the immunogenicity of AdVs vector vaccines. What happens to the viral DNA after delivery is a crucial component of vector safety [36]. It is recognized that a few viral vectors, including lentiviruses-based vectors, can fuse with host DNA and cause genotoxicity. Thus, their application has typically been limited to ex vivo therapies. Because patient follow-up is impossible, these vectors are unsuited for a thorough vaccination campaign [37]. Contrarily, AdVs remain episomal and have not been seen to significantly incorporate into the host genome [32]. Moreover, a high packing capability is also achieved by the loss of the E1/E3 replication genes, enabling the inclusion of huge transgene sequences [38]. Crucially, AdVs’ wide tissue tropism and capacity to strongly induce the expression of the target antigen enables them to be placed among the viral vectors that induce the greatest immune response [39]. This ability to trigger potent immunogenicity has been used to create vaccine candidates for cancer immunotherapies [40] as well as infectious diseases such as Ebola disease [41], AIDS [42], Zika virus disease [43], tuberculosis, and malaria [44]. Recent research has demonstrated the possibility of a recombinant Ad serotype 26 vaccine which generates a Zaire Ebola virus glycoprotein to protect humans from the illness brought on by the Ebola virus [45]. AdVs are easily scalable to fulfil the need for vaccines around the world.

### 2.1. Characteristics of Adenovirus (AdVs)

Commonly used human adenoviruses i.e., Ad5, and Ad26 have a genomic size of 36 kb [46]. The DNA is bound by the hairpin-like structure on both side called inverted terminal repeats (ITR). ITR acts as the self-primer, hence, no additional primase is needed in DNA synthesis [35,47]. It indirectly helps in the multiplication of DNA. ITR also serves a vital part in the amalgamation of the host genome. Besides the ITR, there is another genetic element called a packaging signal that helps in the packaging of a viral transcript [48]. With their role at different stages in viral replication, the viral transcript is classified as early and late. There are four types of early transcript (namely E1, E2, E3, and E4) that help in the representation of non-structural proteins (Figure 2) [49]. Non-structural proteins have a noteworthy role in viral DNA replication. The late viral transcript enciphers the structural components of the adenovirus [50].

### 2.2. Generation of Ad Viral Vector

#### 2.2.1. First-generation Ad Viral Vector

In this generation of adenovirus, E1 and E3 genes are deleted, which disables the adenovirus’ ability to replicate on its own. The cloning capacity is restricted to 8.2 Kb (kilobyte) with higher immunogenicity [51].

#### 2.2.2. Second Generation Ad Viral Vector

These generations of adenovirus were designed to have higher cloning capacity up to 12 Kb with a low level of immunogenicity. Besides E1 and E3, additional regulator genes i.e., E2 and E4 are also omitted [30].

#### 2.2.3. Third Generation (Gutless or High-Capacity Adenovirus Vector) Ad Viral Vector

The Ad vectors of this generation have high cloning capacity up to 36 Kb with negligible toxicity. The vectors are deprived of regulatory genes rather than having genetic elements such as ITR and packing signals [52,53].

### 2.3. Recombinant Adenoviral Vector (rAdVs) Production

The creation of the vector genome generally encompasses the straight cloning recombination or transportation technique in *E. coli* (*Escherichia coli*) cells [54]. There are several methods for the production of the adenovirus and here we will discuss some such methods.

#### 2.3.1. The Traditional Method

**Figure 3:**
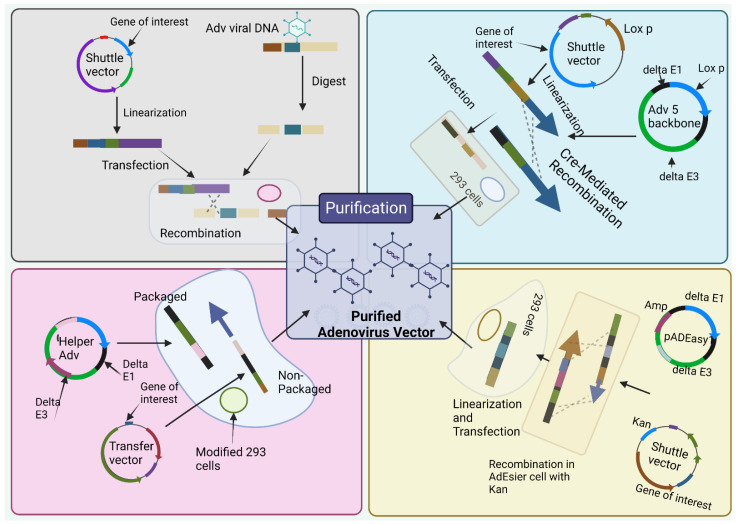
**Production methods for adenoviral vectors.** (**1**) **the traditional method**—recombination in HEK-293 cells. The gene of interest (GOI) is first cloned into a shuttle vector, which contains 5′-ITR, packaging signal, and homologous regions to the adenoviral genome. Adenoviruses are generated in HEK-293 cells through recombination between shuttle vector and adenoviral backbone vector, which is unable to produce virus by itself. (**2**) **Cre/LoxP-mediated recombination.** The GOI is cloned into a shuttle vector that contains LoxP site(s). Cre recombinase-mediated recombination occurs with a LoxP-containing adenoviral backbone vector in vitro or 293-Cre cells, leading to the generation of adenoviruses. (**3**) **The AdEasy system.** The GOI is subcloned into a shuttle vector that contains 5′-ITR and packaging signal, as well as a kanamycin-containing bacterial replication unit flanked with homologous arms. Recombinant adenoviral plasmids are generated through homologous recombination between the linearized shuttle vector and ampicillin-resistant adenoviral backbone vector, such as pAdEasy1, in the bacterial strain BJ5183 cells under kanamycin selection. The resultant adenoviral plasmids are linearized and used for adenovirus production in HEK-293 cells. (**4**) **The use of helper adenovirus for the production of HC**-AdVs (or HD-AdVs, or Gutless AdVs). The GOI is cloned into a transfer vector that contains both ITRs and packaging signals only. Adenoviruses are generated with a helper adenovirus, which will not be packaged due to the deletion of packaging signal in the modified HEK-293 cells, usually through Cre/LoxP or FLP/FRT excision system. (Created using Biorender.com).

The most classical way to obtain E1-deleted AdVs involves the utilization of homologous recombination of two DNA vectors [55]. One of them carries a sequence that maps to the gene of interest at the left end of the adenovirus genome, and the other carries a sequence that overlaps the 3’ viral sequence and continues to the adenovirus genome’s right end (Figure 3A). The whole recombination process occurs in E-1 expressing cell such as HEK-293 cells. This method is rarely used nowadays due to its inefficient adenovirus generation [56]. The production method is labor-intensive and time-consuming.

#### 2.3.2. Cre/LoxP-Mediated Recombination

To overcome the limitation of method 1, Cre-lox site-specific recombination was established which involves three elements [30]. (a) A recombinant adenovirus having two loxP sites (b) a shuttle vector having ITR on the left side, the expression cassette, a packaging signal and loxP site and (c) a 293-Cre cell line that helps to represent Cre-recombinase [30]. When a shuttle vector having a gene of interest and viral DNA transfected together into a 293-Cre cell, an adenovirus genome having the capacity to reproduce and prompt the viral genome is formed due to the intramolecular replication between two loxP sites [30,57]. The formed adenovirus genome cannot be packaged. Another recombination occurs between the loxP site of the formed adenovirus genome and that of the shuttle vector that finally forms the desired recombinant adenovirus (Figure 3B). One of the limitations of this method is the presence of parental adenovirus in the whole preparation method that remains even after multiple passages in 293-Cre cells [30,58]. Hence, this method necessitates cautious confirmation of the identity of the recombinant virus.

#### 2.3.3. The AdEasy System

This method utilizes HEK 293 cells so that the issue of homologous recombination can be reduced by utilizing recombination in microbes such as yeast and bacterial cells [30]. E.g., AdEasy system (Figure 3C) is used to promote the recuperation of the recombinant *E. coli* clone, particularly, by introducing expression cassettes into the E1 region. After purification of recombinant plasmid DNA, it releases the viral chromosome and is relevantly transfected in the cell line [54]. The system largely relies on *E. coli* rather than mammalian cells due to presence of the homologous machinery of bacteria [30,59].

#### 2.3.4. The Usage of Helper Adenovirus for the Construction of HC

To obtainobtain a virion for sufficient packaging, the genome size should fall within the range of 27.7 kb–37 kb [60]. However, for Helper-dependent adenoviral vector (HDAd) genomes involve a noncoding eukaryotic “stuffer” and the adenoviral ITRs and ψ packaging signal [61]. Contrastingly, the helper virus (HV) is E1-deleted and accepts a packaging signal flanked by loxP sites. Succeeding infection of 293-Cre cells, this permits the packaging signal to be removed from the HV genome, rendering the HV genome unpackageable. However, DNA replication is still possible for it and thus complements the replication and encapsidation of the HC-AdV genome in trans. A site-specific recombinant system based on FLP/FRT known as the ‘‘Alternative HC-AdVs’’ production system has been developed and assessed for similar results [62]. While packaging, to some extent HV contamination can be correlated with removal of signal of either the Cre/LoxP or FLP/FRT system, if it was not sufficient. Other methods include AdVac system42, Gateway recombination technology, etc.

### 2.4. Vaccine Design/Process Development for COVID-19

Positive-stranded RNA viruses known as coronaviruses have a nucleocapsid (N) protein that houses the genome and is encircled by the proteins that make up the envelope (E), membrane (M), and spike (S) [63]. Targeting various structural proteins of the coronavirus, several vaccine studies were conducted, though after the SARS and MERS outbreak most of these efforts had come to an end. With the current COVID-19 pandemic, it is crucial that coronavirus vaccine research be restarted. In response to the current epidemic, the first human trials of an mRNA-based vaccine that targets the S protein of SARS-CoV-2 started on March 16th, 2020. The two main approaches for creating the coronavirus vaccine are using entire viruses or genetically modified vaccine antigens that can be administered in a variety of ways [64]. Whole virus vaccines have the potential to stimulate a strong immune response and offer protection from coronavirus infections. Genetically engineered vaccinations that specifically target coronavirus proteins are widely used to increase vaccine safety and effectiveness. N, S, and M proteins from the coronavirus can be administered as DNA recombinant vaccines and viral vector vaccines [65]. The introduction of a transgenic cassette into the adenoviral backbone through homologous recombination or direct cloning is a key component of the adenovirus formulation mechanism [66]. Highly robust and sustained expression of transgenes is maintained through a strong promoter in the transgene cassette [67]. Host proteases have the ability to split the S protein into S1 and S2. The Receptor Binding Domain (RBD) of the S1 subunit is essential for the virus to attach to the target Angiotensin Converting Enzyme-2 (ACE2) receptor [68,69]. The S2 subunit comprises fusion peptide that is in charge for membrane facilitation and viral entry. The S protein is of interest due to the presence of epitopes targeted by neutralizing antibodies in a number of vaccine developments [70]. Within five days of transfection, high-titer adenoviruses were developed under ideal transfection conditions. As a result, combining the AdEasy technology with the rapid adenovirus production and amplification (RAPA) cell line should dramatically accelerate AdV production.

#### Transgene Design

Six adhesive SARS-CoV-2 proteins have been predicted by Vaxign analysis viz. nsp3, nsp8, nsp9, nsp10, 3CL-PRO and S protein. Adhesin is involved in the virus attaching to the host genome and enabling virus entrance into the host genome, and it has a direct correlation to vaccine-induced immunity. S protein was anticipated to be the adhesin in SARS-CoV-2, which matches its key role in viral entry [65]. Designing the gene of interest using the S protein of the SARS-CoV-2 sequence, which corresponds to locations 21,536–25,384 in SARS-CoV-2 isolate Wuhan–Hu-1 (GenBank accession number: MN908947), is the optimal course of action [48,71]. Transgenes can be designed based on the native S protein, modification in S protein or epitopes such as the RBD.

### 2.5. Mechanism of Action for Adenovirus Vector-Based Vaccine

Owing to their nanoscopic structure, it is easy for the viruses to invade the host cell, and such is the case with adenovirus. Rowe, along with his company, first attempted to culture adenoid tissue in a lab more than half a century ago. Since then, many have played their part in adenovirology [72]. The field has seen growth due to its high use as a vehicle to transport foreign DNA into the target cell. In the viral vector vaccine, the vector viruses are weakened or attenuated so they cannot lead to any type of disease [73]. Many types of virus (adenoviruses, adeno-associated viruses, poxviruses, lentivirus, paroviruses, measles viruses, togaviruses etc) have served as vectors, and they can be broadly divided into two categories: replication-defective and replication-competent [74]. Replicating vector vaccines infect cells, which produce prophylactic antigens and new viruses, which can then infiltrate more cells and exhibit immunity. Non-replicating vector vaccines, on the other hand, can generate vaccine antigens but not new virus particles [75]. The first DNA virus to enter demanding therapeutic advancement was adenovirus. By virtue of its genetic stability, well-defined biology, and ease of large-scale production it was considered to be a promising vector for transgene delivery [76].

**Figure 4:**
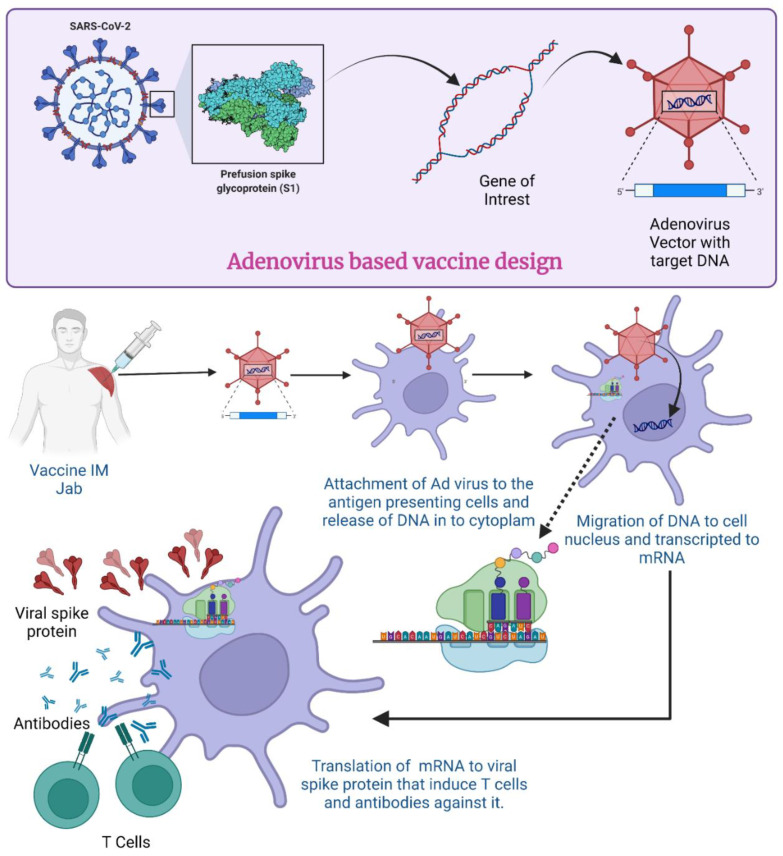
Mechanism of Adenovirus vector-based vaccine for COVID-19. (Created using Biorender.com, accessed on 19 December 2022).

In the case of SARS-CoV-2, when the AdV vector-based vaccine is administered, a virus with the gene for S protein attaches itself to the host cell surface receptor (Figure 4). By the process of receptor-mediated endocytosis, they enter the cell in the form of a vesicle coated by clathrin [77]. Once they are inside the cell, these endosomes accompany vacuoles and undergo fusion with lysosomes, which release acid and digestive enzymes [78]. However, it does not destroy the adenovirus; rather, the endosomes become more acidic which triggers the uncoating of the virus. After the uncoating of the outer membrane, microtubules help transport the viral DNA protein core to the nuclear pore complex [79].

The nuclear pore then disassembles, and DNA is released into the nucleus along with some histones. The DNA now undergoes transcription to produce mRNA inside the nucleus. The formed mRNA is released into the cytoplasm where ribosomes are found. In the translation process, mRNA is attached to ribosomes and produces S proteins. Endosomes inside the cytoplasm chop and break these S proteins into fragments. The fragments of S protein are loaded up on major histocompatibility complex 1 or 2 (MHC-I and -II) which help in the presentation of S proteins on the cell surface [80]. All these events cause activation of natural killer cells, cytotoxic T cells or helper T cells [81]. If an individual becomes exposed to SARS-CoV-2 after receiving the vaccination, their immune system will recognize the known antigens and develop antibodies to fight them [82]. In an investigation with mice and rhesus macaques, administration of Ad5-S-nb2 intramuscularly elicited cell-mediated immune responses (CMI) and systemic S-specific antibodies, as opposed to intranasal administration, which elicited systemic and pulmonary antibodies but a lesser CMI response. Macaques were immune to SARS-CoV-2 after receiving a single injection of Ad5-S-nb2 intramuscularly and intranasally for 30 days [83].

Adenovirus comes in both replicating and non-replicating forms. Replication-competent vectors vary from replication-defective vectors in that only the E3 region is removed. As a consequence, they have a smaller clone potential than replication-defective Ad, which is 3–4 kb. Furthermore, they require doses that are at least 2–3 logs lower compared to those of non-replicating AdV vectors. This dose-sparing impact is attributed to the virus particles’ subsequent reproduction in the cells. It was discovered that a replication-competent adenovirus vector influenza vaccine exhibits long-lasting mucosal and systemic immunity in an investigation on adenovirus-type-4-expressing influenza virus H5 HA (Ad4-H5-Vtn) to test the strength and persistence of the immune response of a replicating vaccine [28].

The vaccine against Ebola virus (ERVEBO) developed by Merck in collaboration with IAVI is another example of a replication-competent vector. The vaccine against replication-competent vesicular stomatitis virus vectors also offers defense against illnesses linked to SARS-CoV-2. It was found to be effective in mice [84] and research is ongoing in humans. These replication-competent viral vectors help in improving transgene immunogenicity [85] and require lower doses to confer persistent immunity [86].

*Discussion of the outcome of the virus entering non-antigen presenting cells, the possible systemic release of the encoded antigen.*

All of the body’s nucleated cell types are non-professional antigen-presenting cells. To display endogenous peptides on the cell membrane, they bind an MHC class I molecule to beta-2 microglobulin [87]. The infected cells exhibit viral peptides inside MHC class I antigens throughout the course of the viral infection cycle. Viral peptides from class I will activate CD8+ T lymphocytes, which have the ability to lyse virus-infected tissue cells. Activated CD8+ T cells multiply, develop, and become virus-specific effectors and memory T cells. Professional antigen-presenting cells (dendritic cells, macrophages, and B-lymphocytes) expose viral peptides to CD4+ T cells in the early stages of infection via MHC class II molecules [88]. The cellular introduction of a recombinant viral genome (cDNA) including a promotor sequence, the gene encoding the antigen, and a poly-A tail, is the basis for the functioning of adenoviral vector vaccines. Upon intramuscular delivery, muscle cells become infected, present a processed antigen via MHC class I, and begin to produce a viral antigen in order to activate antigen-presenting cells and elicit an immune response. This mechanism of action has the benefit of activating both the innate and adaptive immune systems, which will cause a humoral and cellular response [89].

## 3. Adeno Viral Vector-Based Vaccine-Based Platform for COVID-19: Intramuscular Injection

As of 22 April 2022, more than 507 million individuals have been infected and among them 6.2 million lost their lives during this coronavirus pandemic [90]. Different vaccine strategies have been established to provide a safe and efficacious immune response. Among them, viral vector-based vaccines have shown a good safety and efficacy profile which mainly includes 14% non-replicating and 5% replicating viral vector-based vaccines [91].

University of Oxford and AstraZeneca manufactured AZD1222, which consists of a ChAdOx1 adenoviral vector and the gene of the S protein of coronavirus administered via the IM route. In AZD1222, the coding sequence has not been changed in order to stabilize the expressed S protein in the prefusion conformation; instead, the SARS-CoV-2 S immunogen is expressed in the vaccine in the trimeric prefusion conformation [92]. The Serum Institute of India’s version of AZD1222 is named Covishield. Around the world, eight phase I, 30 phase II and 11 phase III trials have been registered for AZD1222. Whereas for Covishield, one phase II and one phase III trial have been registered [93].

A phase ½ single blind, randomized controlled trial carried out with 1090 participants in the 18–55 age group showed good safety and immune response [94]. A phase II/III trial was conducted involving 160 participants in the 18–55 age group, 160 participants in the 56–69 age group and 240 participants in the 70 years and older age group receiving the vaccine via the IM route. The result of the phase II trial showed injection site pain, mild fever and headache [95]. A phase III trial involving 23,848 participants above the age of 18 years receiving this vaccine to determine safety and efficacy. Four randomized phase III trials conducted in the UK, Brazil, and South Africa yielded positive safety findings and 62.1% effectiveness [96].

Several medicinal agencies such as The Australian Therapeutic Goods Administration and The European Medicines Agency (EMA) have permitted the Covishield vaccine [97]. Additionally, the World Health Organisation (WHO) has approved the vaccine for an Emergency Use Listing. As per the WHO report, AZD1222 has been approved for immunization in 125 countries, and Covishield in 46 countries [98]. The vaccine was first authorized for use in the UK immunization program on 30 December 2020, and the first vaccination outside of a study was given on 4 January 2021 [99]. The vaccine is 76.0% efficient in eliminating symptomatic COVID-19 commencing 22 days following the first dose, and 81.3% efficacious after the second dose, according to studies done in 2020 [100]. Additionally, according to a Scottish study, the vaccine is 81% effective against the Alpha variant and 61% effective against the Delta variant for symptomatic COVID-19 infection after the second dose [101].

The Gamaleya Research Institute developed Sputnik V, or Gam-COVID-vac, which consists of Ad26 and Ad5 vectors with S protein genes of SARS-CoV-2. The Sputnik V vaccine from Gamaleya, which encodes natural S, does not appear to use 2P spike mutations [102]. Phase I/II trials conducted with 76 participants aged 18–60 years showed good safety and immune profile [103]. Phase III trials showed acceptable tolerability and 91.6% efficacy, and the vaccine was approved in Russia [104]. Based on the preliminary results of Phase I/II studies released on 4 September 2020, the vaccine was accepted for distribution in Russia as of April 2020 and thereafter in 69 other countries [105]. Despite this, the vaccine is not authorized by WHO.

Janssen Pharmaceutical prepared the Ad26Cov2-S vaccine using an Ad26 vector including the genes of coronavirus. It expresses the S protein and contains K986P and V987P alterations (2P) in a loop that abuts the S2’ membrane fusion machinery’s core helix. Higher titers of neutralizing antibodies are produced as a result of the alteration, which traps the spike in a prefusion conformation. It also features a deletion at the location where furin cleaves [48]. The vaccine is administered via the IM route in a single 0.5 mL dose. A phase I/II study on 1045 participants divided into age groups 18–55 and above 65 demonstrated acceptable safety and immunity profile. The Janssen COVID-19 vaccine showed 66% efficacy in preventing severe COVID-19 [106].

CanSino Biologics developed Convidecia (AD5-nCOV) using an Ad5 vector with genome of S protein of SARS-CoV-2. It is a single dose vaccine. A phase I dose escalation trial was carried out in healthy volunteers, 108 of which were in the 18–60 age group, and results showed a safe and good tolerability profile [107]. Phase II dose escalation studies performed in 508 participants above 18 years of age demonstrated good immunogenicity and tolerability [108]. The results of the Phase III trial revealed that the vaccination was 65.7% effective in preventing intermediate COVID-19 symptoms and 91% effective in treating severe disease [109]. Two doses of the nasal spray version of the vaccine Convidecia led to identical immunological antibody responses to the previous one-dose regimen, according to a Phase I trial report published in *The Lancet* [110]. Apart from Ad vector-based vaccines, the MVA-SARS-2-S vaccine, consisting of a Modified Vaccinia Virus vector expressing the S protein genome of coronavirus, developed by University of Munich showed good tolerability and safety profile under a Phase 1 clinical trial [111]. City of Hope Medical Center, NCI, developed COH04S1, which mainly contain a synthetic modified vaccinia Ankara vector encoding S protein. A phase I dose escalation study to evaluate safety and efficacy of vaccine has been carried out [112]. TMV-083(V591), developed by Institute Pasteur, Themis Bioscience GmbH Coalition, consists of a live attenuated rMVV vector encoding surface glycoprotein of coronavirus. It is currently undergoing administration of a Phase I placebo-controlled, randomized trial to assess the COVID-19 vaccine’s immunogenicity and safety [113]. Table 1 highlights the EUA Ad vaccines for COVID-19.

## 4. Ad vector-Based Vaccine Platform for COVID-19: Intranasal Delivery

Intranasal vaccination can be considered as the most attractive way of vaccination from all mucosal routes owing to greater patient compliance [120]. A strong IgG response is produced by the IM injection, which is considered to protect the lower respiratory tract, but not the necessary epithelial cell-based IgA responses to safeguard the upper respiratory tract [121]. A vaccination that triggers sterilizing immunity in the upper airway will be ideal for preventing the spread of viruses. In addition to offering protection from symptomatic illnesses, nasal vaccine administration could inhibit infected individuals from transmitting the virus [122]. Intranasal vaccination is a potential approach since it ties the typical way of infection, is simple to administer, and also has the probability to obtain a significant market share in the future. Intra-nasal vaccination produces potent neutralizing antibody responses as well as mucosal IgA and T cell responses that practically completely eliminate SARS-CoV-2 infections in both the upper and lower respiratory tracts. A nasal spray, as opposed to injections, is painless and tempting to people who are afraid of needles [121].

The method provides complete sterility in the nose that is the foremost entrance point for the virus. Trained personnel are also not required; self-administration is possible. Along with that, the method is adapted to affiliate global needs. Several studies have shown that the viral S Protein is the major cause of viral attachment and engaging the cell surface receptor ACE-2 [123,124,125]. ACE-2 is predominantly found in the nasal epithelium, and to a lower degree in alveoli [126]. Therefore, sterilization of the nasal route prevents the infection. To date, a vast number of adenovirus vector vaccines developed against several infections. For COVID-19 also, some innovators’ products are under investigation for intranasal vaccine delivery. Table 2 summarizes intranasal vaccine for COVID-19 under development based on Ad vector platform.

### 4.1. Altimmune

Altimmune has developed AdCOVID™, which is characterized as a needle-free intranasal single dose non-replicating Ad-5-vectored vaccine. AdCOVID™ strongly generates humoral and cellular immune response [129]. Generally, by potential IgG serum neutralizing activity and through a 29-foldrise in mucosal IgA in the airway track, this vaccine candidate has crossed the preclinical stage and is under phase 1 clinical trial (NCT04679909). Antigen-specific CD8^+^ killer T cells are detected in the lungs that confirm cellular immunity. Data from clinical trials, however, has shown that AdCOVID^TM^ did not sufficiently activate immunity. As a result, Altimmune, Inc. announced that AdCOVID^TM^ development will no longer be pursued.

### 4.2. AstraZeneca

ChAdOx1/AZD1222 is formulated as an IM injection and has also been assessed for the intranasal route in non-human primates [130]. The outcome of both routes is close and shows significantly high IgG titers. After successful vaccination when compared to control animals, it suggested downregulating the viral load in lungs and significant absence of virus in BAL fluid. Therefore, it demonstrates that intranasal vaccination shows inhibition of viral activity in the lower respiratory tract. The four immunized monkeys were subsequently challenged to SARS-CoV-2, together with four untreated rhesus monkeys. The immunized monkeys had less virus in their nostrils and lung tissue, and none had pneumonia symptoms, which three untreated monkeys developed [131]. In 54 healthy participants, the University of Oxford is testing the efficacy of the intranasal ChAdOx1 vaccine (NCT04816019).

### 4.3. Bharat Biotech-Washington University

The advance of the SARS-CoV-2 vaccine (BBV154) relies on chimpanzee adenovirus as has been established by Washington University, School of Medicine in association with Bharat Biotech. For defensive action at the lower and upper respiratory tract a single dose intranasal vaccination exhibited higher neutralizing antibody action [132]. The intranasal vaccination experiment on 12 non-human primates resulted in high Anti-RBD, Anti-S, IgG, as well as potential cellular immunity and shows significant preventive action in the lungs and BAL fluids. A phase I randomized multicenter clinical study of BBV154 is currently being conducted with 175 individuals who will receive one or two IN doses of vaccination (NCT04751682).

### 4.4. CanSino Biologics Inc./Beijing Institute of Biotechnology

In the early 2020s, CanSino Biologics Inc. and the Beijing Institute of Biotechnology developed Ad5-nCoV, a human adenovirus serotype 5 vaccine. It is an Ad5-derived first-generation E1/E3-deleted vector that expresses the whole SARS-CoV-2 spike glycoprotein in full length. In mice and ferrets, where SARS-CoV-2 infection exclusively occurs in the upper respiratory tract as opposed to the lungs, Ad5-nCoV’s efficacy was examined. The upper and lower respiratory tracts of mice were totally shielded from SARS-CoV-2 when the intranasal (IN) and intramuscular (IM) injection routes were contrasted. However, because of issues with IN administration in individuals with asthma, IM was chosen for Ad5-nCoV immunization in early human clinical research. [133]. Ad5-nCoV seems to be well tolerated and possesses the ability to produce humoral and cellular immunity, according to the phase 1 and phase 2 findings. An initial study from February 2021 revealed that, following a single dose, Ad5- nCOV avoided 90.07% of serious diseases and 65.28% of symptomatic cases [134].

### 4.5. Ad Vaccines for SARS-CoV-2 Variants

The emergence of new vaccinations against potentially more virulent SARS-CoV-2 strains has sparked new research attempts. Concerns about the potential return of a viral variation resistant to the protection generated by the currently available vaccinations are particularly highlighted by the D614G spike protein mutation [135,136]. The hAd5-S-Fusion+ ETSD vaccine was developed by ImmunityBio, Inc. (Culver City, CA, USA) and NantKwest Inc. (San Diego, CA, USA) to administer both the S-Fusion as well as N-ETSD proteins, which are improved versions of the SARS-CoV-2 spike and nucleocapsid proteins, respectively. Given that the nucleocapsid protein is conserved in all known SARS-CoV-2 variations, vaccinations containing this antigen may continue to provide defense against newly discovered variants [137]. The actual vector is derived from a second-generation Ad that has had the E3, E1 and E2b genes eliminated and has already been employed in the existence of pre-existing immunity to Ad5. Preclinical studies demonstrate that vaccination against the spike and nucleocapsid proteins of the SARS-CoV-2 virus generated humoral as well as cellular immune responses in a mouse model [138].

The Omicron variant, which carries mutations, has recently increased dramatically, raising worries about immune evasion even in those who have received all of the recommended vaccinations. The latest research has looked at the effectiveness of booster vaccinations given after CoronaVac/AZD1222 prime to produce T-cell activation and neutralizing antibodies (NAbs) towards BA.1 and BA.2 omicron. A number of 167 subjects were recruited to obtain BNT162b2, AZD1222, or mRNA-1273 as a third dosage after being immunized with heterologous CoronaVac/AZD1222 for 4–5 months. Total interferon responses, reactogenicity, immunogenicity, and the NAb titers towards BA.1 and BA.2 were all measured. After 4–5 months from the administration of the heterologous CoronaVac/AZD1222, a noteworthy loss in neutralizing potency to the omicron variant was discovered. Without any severe adverse effects, reactogenicity was mild to moderate. A dramatic rise in associating antibodies and neutralizing activity against the delta and omicron variants was seen after a booster immunization. The greatest titers for omicron BA.1 and BA.2 neutralization were obtained following mRNA-1273-boosted dose, then BNT162b2 and AZD1222. In contrast to those who received AZD1222 as a booster, those who received messenger RNA (mRNA) vaccinations acquire a T-cell response to the spike protein [139].

Over the last few days, novel subvariants of Omicron BA.5, including BA.5.1.7 and BF.7, were found throughout China. The two novel subvariants are extremely contagious and spread quickly.

The research shows that inhaling the COVID-19 vaccine after receiving two doses of the inactivated vaccine raises levels of neutralizing antibodies against a number of variants, including BA.5. It was published on October 5, 2022 in the prestigious infectious diseases journal *Emerging Microbes and Infections*. Aerosolized vaccine injection in the respiratory mucosa can produce local mucosal IgA antibodies and a higher level of antibody count against the Omicron variations in blood, which may effectively treat the Omicron variant BA.5 [140].

## 5. Challenges to Adenoviral Vector Use for Vaccine Delivery

The potential for interaction between the viral gene and the host genome needs to be carefully examined before entry and throughout clinical progress. Most of these worries about safety are considerable, and they could cause clinical research to be delayed in the event of a pandemic [81,141]. It has been proposed that several variables, such as the amplitude and frequency of vaccine antigen synthesis post-vaccination, lead to the increased immunogenicity of certain Ad vectors [33]. Moreover, it has been indicated that the elevated occurrence of Type I interferons by certain Ad vectors may impede transgene expression patterns, inhibiting successive immune functions. As a result, it is best to induce stable, but not excessive, activation of innate signaling. The quantity and availability of innate immune cells and/or antigen-presenting cells at the injection site affect the initial innate immune responses to the Ad vaccine, which affects the outcome of the adaptive immunity [33,69,74]. Implementing tailored and specialized Ad vaccine platforms for certain diseases will require a better knowledge of how various features of the host immune response to AdVs vectors relate to their immunological potential.

### 5.1. Pre-Existing Immunity

Pre-existing immunity is one of many challenges that need to be tackled by all Ad vector-based vaccine manufacturers. The unique serotype of adenovirus that is hardly exposed to humans can be an option for those who are infected by adenovirus [142,143,144]. If pre-existing immunity is present, the viral vector-based vaccine will be less effective. Measles virus (MV) is an enveloped virus which contains a single-stranded RNA genome. MV virus produces both humoral as well as cellular immune responses against transgene [145]. When one introduces the transgene into the virus, the introduction is quite difficult. The solution to this challenge is the introduction of a transgene in a virus at different positions [146]. In studies in mice and macaques, pre-existing immunity is not a barrier. However, in human studies, pre-existing immunity decreases vaccine efficacy [85,147]. Some solutions to the problem of pre-existing immunity are as follows:

#### 5.1.1. Use Rare Viruses as a Vector

Ad5 is a common type of AdV. If one uses Ad5 for vaccine production, Ad5-based vaccines will be less effective. Adenovirus is a virus that naturally exists and mainly infects our upper respiratory tract, rendering individuals with some immunity against adenovirus [148,149]. Thus, using a rare adenovirus such as Ad26 is preferable to avoid such existing immunity-related issues. However, Africans have 90% immunity against Ad26 [150]. Another solution is the production of an adenovirus vector-based vaccine that infects non-human species. In the case of AdVs, usually, a chimpanzee adenovirus vector is used as a human does not neutralize such adenovirus vectors. Chimpanzees possess AdC68 species. Since the AdC68 virus enters the cell via connecting with chimeric antigen receptors (CARs), the response will be the same as with the AdHu5 virus even though humans do not possess neutralizing antibodies to this virus [151]. While the AdC6 virus is not cross-neutralized by antibodies to the AdC68 virus, the AdC7 virus exhibits partial cross-reactivity for virus-neutralizing antibodies to AdC68, suggesting that the two viruses may belong to the same serotype [152].

#### 5.1.2. Use Different Virus Vectors for Priming and Booster/Additional Dose

Sputnik V COVID-19 vaccine uses Ad26 for the priming dose and Ad5 for boosting/second dose as a vector [150]. However, the AstraZeneca-Oxford COVID-19 vaccine uses a chimpanzee Ad vector and each dose contains the same vector [94]. In mice, if an Ad5 vector is coated with polyethylene glycol or encapsulated into alginate microspheres, transduction increases in presence of pre-existing immunity [153,154,155]. The emphasis has since shifted to determining the pros and cons of heterologous boosting treatments, wherein a primary immunization sequence of one or two doses is accompanied by a third dose administered at least 3 to 6 months afterward with a separate platform vaccine for COVID-19 [156].

#### 5.1.3. Different Routes of Immunization

The best way to overcome pre-existing immunity is oral immunization. However, this only produces a response against neutralizing antibodies and it fails to produce a response against T cells [157]. An intranasal administration is the best option for COVID-19 vaccination [158]. An intranasal booster dosage after the IM vaccine administration activates memory B and T cells in the upper airways to provide mucosal immunity to prevent viral propagation and infections. This produces a long-lasting systemic IgG response. The second-generation intranasal vaccinations are anticipated to help in lowering the transmission rate, improving our power to eliminate this virus. The first-generation intramuscular vaccines aid in controlling virus spread [121].

#### 5.1.4. Modification in Vector

The lentivirus vector has a tropism for both dividing and non-dividing cells [159]. By making some modifications, one can overcome pre-existing immunity problems. By the addition of vesicular stomatitis virus glycoprotein in the lentivirus, pseudotype lentivirus is formed, giving increased safety [160]. A lentivirus vector with a tetracycline promoter increases the expression. Min-Wen Ku and colleagues have developed a lentivirus vector-based COVID-19 vaccine for intranasal immunization and evaluated it in golden hamsters as a part of the pre-clinical evaluation [161]. Results show that it produced a sufficient amount of spike glycoprotein-neutralizing antibodies to provide the host with immunological protection.

### 5.2. Heterologous Immunity

Some observations show that cross-reactivity is present between non-related pathogens. This is called heterologous immunity. Heterologous immunity can either increase or decrease the response of a vaccine. Heterologous immunity mainly forms due to cross-reactivity of T-cells or neutralizing antibodies [162]. Heterologous immunity exists between adenovirus and hepatitis C. Both adenovirus and hepatitis C have the same structure with different epitopes. This cross-reactivity occurs in both mice and humans. Adenovirus vector-based vaccines induce homologous immunity and humoral immunity against multiple hepatitis C viruses. These vaccines have cross-reactive T cells, and reduce the chance of SARS-CoV-2 infection [163,164,165]. Jiaojiao Liu and Coworkers showed encouraging vaccine efficacy findings against the SARS-CoV-2 alpha and beta variants using the heterologous prime-boost method and vaccines made from chimpanzee adenoviruses [166].

### 5.3. Thrombocytopenia

Because of reports of unusual blood clots, such as heparin-induced thrombocytopenia (HIT), linked to the COVID-19 vaccinations, the introduction of the Oxford/AstraZeneca and Johnson & Johnson vaccines was halted in many countries in the middle of 2021. However, the vaccination was resumed after a review of the data, except in Denmark, which is the only country where the Oxford/AstraZeneca vaccine is still not being administered [167,168]. One observation showed availability of antiplatelet factor 4 (PF4) antibodies among individuals with Vaccine Induced Thrombocytopenia (VIT) [169]. Heparin or similar structures can be bound by PF4, and in certain people this can trigger an autoimmune response against the structures. This results in a sequence of subsequent events that finally result in thrombocytopenia [170]. Numerous theories have been proposed in an effort to explain the connections between Ad vaccines, PF4 and VIT. An initial research revealed that the SARS-CoV-2 spike and anti-PF4 antibodies do not bind to one another, likely ruling out the hypothesis that the vaccination antigen is the cause of VIT [171]. According to one study, the Oxford/AstraZeneca vaccine potentially interacts with PF4 and triggers immunological responses that are pro-inflammatory at the injection site [172]. Furthermore, studies revealed that the Oxford/AstraZeneca vaccine has high concentrations of protein-based contaminants, notably heat-shock proteins [173]. Assuming that each vaccination has a somewhat different purifying procedure, such contaminants also may lead to immunologic responses to the vaccine or may clarify variations in VIT rates between both the major licensed Ad vaccines. Concerning the Ad vaccination VIT, several uncertainties remains. Fortunately, VIT appears to still be a rare problem, and clinical therapy of the illness has swiftly advanced, with effective treatments of one patient documented [22]. Hopefully, more appropriate effective treatments and more awareness can help prevent any more unfortunate fatalities. Table 3 explains the safety concerns of intramuscular vaccines and intranasal vaccines.

## 6. Booster Dose Strategy

Coronaviruses mutate at a slower rate than influenza viruses. The existing vaccinations appear to be significantly less effective against many of the new viral variants, this does not necessarily imply that vaccinations will need to be modified each year as they do with flu [184]. A booster dose is usually administered by injecting individuals with an extra dose to the fully vaccinated individual [185]. In the case of COVID-19, it was advised to receive two injections of the majority of vaccinations; however, six months later, there was a reduction in immunity, so doctors now advise a booster dosage, which is the third injection [186,187]. After a period, levels of circulating antibodies that may attach to viruses and limit their capacity to infect humans tend to drop, implying that protection ends [188,189]. The better route of administration and frequency of booster dose administration is under investigation. The Oxford-AstraZeneca vaccine would be less effective against the beta variant, and the prospect of a “booster” dose for persons who have previously been vaccinated toward COVID-19 has been mentioned [190].

The technology that underpins these vaccinations is also distinct. Unlike conventional influenza vaccines, which are created by cultivating flu viruses in chicken eggs and then extracting and deactivating them, the authorized COVID-19 vaccines are created by inserting the genetic instructions for generating the coronavirus spike protein into the human cells, or into an innocuous adenovirus, which further transports this information into our cells [68,69,121]. In principle, fine-tuning these vaccinations should be faster and easier than isolating and growing vast quantities of viruses in chicken eggs [191]. Numerous COVID-19 vaccines have been shown to be secure and effective when administered as a booster dose [192]. Because these are novel vaccination technologies, injecting someone with an updated dosage of vaccination on top of an old vaccination has not been well researched [69]. In theory, there is no reason to believe it will not work, but additional safety studies may be necessary. Everyone believes that obtaining two doses of the presently offered COVID-19 vaccinations is likely to be preferable to receiving only one. Mostly in the case of the Pfizer/BioNTech vaccine, for example, while antibodies against the virus were discovered 12 days after the first ‘priming’ dosage, the response was greater after the second dosage [193]. Following two doses of either the AstraZeneca or Pfizer vaccinations, the Cov-Boost investigation looked at the use of seven different vaccines as boosters: AstraZeneca, Curevac, Johnson & Johnson (Janssen), Moderna, Novavax, Pfizer, and Valneva. The study showed that all vaccinations (apart from Curevac, which was stopped) enhanced the immune response. However, the concentration of antibodies varied substantially depending on the vaccine combination [194,195,196]. As per Andrew and colleagues [187], “The relative effectiveness against symptomatic disease 14–34 days after a BNT162b2 or mRNA-1273 (Moderna) booster after a ChAdOx1-S (Astrazeneca) and BNT162b2 as a primary course ranged from around 85 to 95%. Absolute VE ranged from 94–97% and was similar in all age groups. Limited waning was seen 10+ weeks after the booster. Against hospitalization or death, absolute effectiveness of a BNT162b2 booster ranged from around 97% to 99% in all age groups irrespective of the primary course with no evidence of waning up to 10 weeks.” Similar to this, a phase 2 trial that involved 18 sites and three different groups and was blinded, multi-center, randomized, controlled, and intended to assess the safety and immunogenicity of seven COVID-19 vaccines as a third dose (booster) after two doses of ChAdOx1 nCov-19 or BNT162b2 revealed significant differences in humoral and cellular responses. Vaccine accessibility will also affect policy decisions regarding booster vaccination [192].

The Omicron version is naturally resistant to being neutralized by plasma from both recovering patients and those who have received one of the four COVID-19 vaccinations that have been widely rolled out (Pfizer, AstraZeneca, Moderna, and Johnson & Johnson). Even serum from those who had been immunized and boosted with mRNA-based vaccines had significantly reduced neutralizing antibody efficacy against the Omicron variant [197]. There is surely the need for a booster dose for the omicron variant in elderly patients to provide sufficient immune protection [17]. According to Rolando Pajon and co-workers [198], “a booster dose of mRNA-1273 vaccine was associated with neutralization titers against the omicron variant that was 20.0 times higher than those assessed after the second dose of vaccine, and these titers may substantially reduce the risk of breakthrough infection.”

### Importance of Anti-SARS-CoV2 Vaccination in Patients with Autoimmune Diseases

Patients with autoimmune diseases and primary/secondary immunodeficiency disorders including rheumatoid arthritis, psoriasis, and inflammatory bowel diseases would be significantly impacted by the COVID-19 pandemic. Several anti-COVID-19 immunizations are safe and effective in treating individuals with immune-compromising diseases, including those using medications that influence the immune system, according to numerous clinical experimental studies [199]. Inactivated COVID-19 or subunit COVID-19 vaccines may respond lower risk of complications in patients with autoimmune diseases. R. Hayward et al. conducted clinical studies in RA patients following anti-COVID-19 vaccines demonstrating that there was a minimal need to be concerned about the safety of shots given to rheumatoid arthritis patients. According to the statistics, those patients did not need any prospective treatments, which would have reduced their likelihood of developing a disease flare [200]. Even though the number of adverse outcomes varied among the various COVID-19 shots, all were equitably accepted in RA patients and both were comparable to healthy subjects, ensuring continuity over the safety of COVID-19 vaccination, according to another study conducted by R. Naveen et al. on short term online assessment of COVID-19 vaccination-related adverse events in rheumatoid arthritis patients [201]. Table 4 highlights the difference between viral vector vaccine and RNA vaccines.

## 7. Concluding Remarks and Future Prospects

Perceiving all the coronavirus outbreaks in 21st century (“SARS in 2002, MERS in 2012, and COVID-19 in 2019”), this can be assumed that similar outbreaks are likely to occur in the future. However, to limit the current COVID-19 outbreak, the speedy development of a safe, reliable, and effective vaccine is crucial along with efficacious treatment [10]. Regardless of age, an ideal vaccine would be able to elicit an effective immunogenic response with minimal antigen dose against several viral mutants of the same disease with no or minimal side effects.

Viral vectors can produce the viral protein of interest with high titers, which serve as the foundation for advanced vaccine development. For identifying virulent antigens, varieties of viral vector expression systems were explored by the researchers. Non-replicating Ad vectors are promising vaccine carriers for infectious diseases due to better yield, cGMP friendly manufacturing processing, safe to use, high efficacy during clinical trials, and manageable shipping and storage procedures. Ad26Cov2.S and/or Ad5-nCoV single-dose vaccines use an inactivated common cold virus as a vector for delivering the viral spike protein gene into the cells, which is subsequently expressed by the host cell with a cascade of immune reactions post-protein expression. Apart from that, they are a better carrier option for mucosal vaccine delivery that provides localized immune protection to the individual and helps in the prevention of viral spread [121].

In India, as per data from the Ministry of Health and Family Welfare, the total number of vaccinations until 9 February 2022 was 1.58 billion. As per the WHO source as of 9 February 2022, 28 vaccines are approved for use. Among these approved vaccines six candidates are Ad vector-based vaccines. Furthermore, a good number of vector-based vaccines are in the clinical and preclinical stages of research. This vaccine platform will be crucial in mitigating the current pandemic situation. Investigational outcomes in non-human primates showed clear favorable protection of virus assembly and shedding resulting in the generation of mucosal immune reaction (sIgA) in the human respiratory tract, as well as sturdy systemic and humoral immune responses. An IM prime accompanied by a booster immunization would almost certainly result in a more well-rounded immune reaction, along with viral replication inhibition (or strong reduction) in the human respiratory tracts. In short, Ad viral vector-based vaccine platform has a lot of potential to be delivered by various routes of administration to generate sufficient immune protection against different viral variants. Various foreign funding agencies, on the other hand, should galvanize and step up to support the vaccine advancement program by overcoming obstacles and amassing the COVID-19 vaccine.

## Figures and Tables

**Table 1:** EUA-licensed adenoviral vector-based vaccines for COVID-19 *via* intramuscular route.

Innovator	Vaccine	Vector Type	Phase	Formulation	References
AstraZeneca/University of Oxford	AZD1222 (Covishield and Vaxzevria)	ChAdOx1	III NCT05293665 NCT04973449 NCT05236491 and 14 more trials are ongoing.	**Active Component:** Replication-deficient chimpanzee Adenovirus vector containing S protein gene of coronavirus. **Excipients:** L-Histidine, L-Histidine hydrochloride monohydrate, magnesium chloride hexahydrate, polysorbate 80, ethanol, sucrose, sodium chloride, disodium edetate dihydrate, water for injections	[94,95,96]
Gamaleya Research Institute/Acellena Contract Drug Research And Development	GamCOVID-Vac (frozen) and GamCOVID-Vac Lyo (lyophilized) (Sputnik V)	rAd26 and rAd5	III NCT04640233 NCT04564716 NCT04530396 NCT04642339 NCT04656613 NCT04954092	**Active Component:** Replication-deficient rAd26 and rAd5 vector with S protein gene of coronavirus. **Excipients:** Tris(hydroxymethyl)aminomethane, sodium chloride, sucrose, magnesium chloride hexahydrate, disodium EDTA dihydrate (buffer), polysorbate 80, ethanol 95%, water	[114,115]
Janssen Pharmaceutical	Ad26Cov2-S (JNJ-78436735)	rAd26	III NCT05047640 NCT04505722 NCT04614948 NCT04838795 NCT05220397 and 3 more trials are ongoing.	**Active Component:** Replication deficient rAd26 vector, encoding a stabilized variant of the SARS-CoV-2 Spike (S) protein **Excipients:** Ethanol, polysorbate-80, 2-hydroxypropyl-β-cyclodextrin, citric acid monohydrate, trisodium citrate dehydrate, sodium chloride	[106]
CanSino Biologics/Beijing Institute of Biotechnology	Ad5-nCoV (Convidecia)	rAd5	III NCT05169008 NCT04540419 NCT04526990	**Active Component:** Replication deficient rAd5 vector with S protein gene of coronavirus.	[107,108,109]
Gamaleya Research Institute/Acellena Contract Drug Research And Development	Gam-COVID-Vac	rAd5	II NCT05248373; Phase III is ongoing	**Active Component:** Replication deficient rAd5 vector with S protein gene of coronavirus. **Excipients:** Tris(hydroxymethyl)aminomethane, sodium chloride, sucrose, magnesium chloride hexahydrate, disodium EDTA dihydrate (buffer), polysorbate 80, ethanol 95%, water	[116,117]
Gamaleya Research Institute/ Acellena Contract Drug Research And Development	Sputnik Light	rAd26	III NCT04741061 and 5 more trials are ongoing.	**Active ingredients:** Replication deficient rAd26 containing the SARS-CoV-2 protein S gene **Excipients:** Tris (Hydroxymethyl) amino methane, sodium chloride, sucrose, magnesium chloride hexahydrate, EDTA disodium salt dehydrate, polysorbate, ethanol, water for injection	[118,119]

**Table 2:** Intranasal vaccine for COVID-19 under development based on Ad vector platform.

Vaccine Name and Innovator	Ad Vector Type	Phase	Clinical Trial Number	Nasal Delivery Device	Remarks
AZD1222 (ChAdOx1) And The University of Oxford (UK) with AstraZeneca (Cambridge, UK)	Non-replicating rChAd vector	I	NCT04816019	Mucosal atomization Device (MAD Nasal™)	rAd vector taken from chimpanzee In animal study with hamster, the viral load is low in the intranasally administered hamster as compared to control group [127].
ChAd-SARS-CoV-2-S/BBV154 And Bharat Biotech (Genome Valley, India)-Washington University (USA)	Non-replicating rChAd vector	I	NCT04751682	Currently, Pipette droppers	rAd vector taken from chimpanzee Intranasal vaccination with ChAd-SARS-CoV-2-S provided long-term defense against both previous and developing SARS-CoV-2 variants in mice [128].
AdCOVID^TM^ And Altimmune	Non-replicating Ad5 vector	I	NCT04679909	Pipette droppers	Developed from Ad vector that encrypts the RBD. The study is currently on hold.
SC-Ad6-1 And Tetherex Pharmaceuticals Corporation	Non-replicating single cycle rAd6 vector	I	NCT04839042	Direct inoculation into the nose	Developed from single- cycle adenovirus against SARS-CoV-2. During Phase I trial, there are issues on safety and tolerability of the intranasal vaccine were observed up to 106 days following vaccination.
Ad5-nCoV And CanSino/Beijing Institute of Biotechnology (China)	Non-replicating rAd5 vector	I/II	NCT04840992	Aerogen Ultra Device	Developed from adenovirus type-5 vector Two doses of aerosolised Ad5-nCoV induced neutralizing antibody responses comparable to a single intramuscular injection [110].

**Table 3:** Safety Concerns of Intramuscular vaccines and Intranasal vaccines.

Adverse Event	Vaccine Reported	Explanation	References
Guillain-Barré syndrome (GBS)	J&J/Janssen	In the uncommon illness known as GBS, the immune system of the body damages nerve cells, leading to muscular weakness and occasionally paralysis. Men 50 years of age and older make up the majority of GBS cases reported.	[174]
Capillary leak syndrome (CLS)	mRNA COVID-19 Vaccines	The COVID-19 vaccination-related adverse event after immunization (AEFI) known as capillary leak syndrome (CLS) has recently appeared. Increased capillary permeability in CLS, a rare disorder that mostly affects the upper and lower limbs, causes hypoalbuminemia, hypotension, and edema.	[175]
Anaphylaxis	Pfizer-BioNTech or Moderna (mRNA COVID-19 vaccines), A tetravalent cold-adapted live-attenuated influenza vaccine (LAIV) produced by Medimmune/AstraZeneca and Nasovac^®^	Approximately five incidences of anaphylaxis have been reported after receiving the COVID-19 vaccine for every million doses of the vaccine. Any sort of immunization might result in anaphylaxis, a severe allergic response.	[176,177,178]
Thrombosis with thrombocytopenia syndrome (TTS)	J&J/Janssen, Pfizer-BioNTech or Moderna (mRNA COVID-19 vaccines)	Approximately four incidences of thrombosis with thrombocytopenia syndrome (TTS) following J&J/Janssen COVID-19 vaccine have been reported per million doses given. TTS is an uncommon but dangerous adverse effect that results in low platelets and blood clots in big blood arteries.	[178,179]
Myocarditis and pericarditis	Pfizer-BioNTech or Moderna (mRNA COVID-19 vaccines)	Pericarditis is an inflammation of the heart’s outer membrane, whereas myocarditis is an inflammation of the heart muscle. The majority of people who had myocarditis or pericarditis after receiving the COVID-19 vaccine reacted favorably to treatment, rest, and improved swiftly. The majority of instances, notably in male teenagers and young adults, have been linked to Pfizer-BioNTech or Moderna (mRNA COVID-19 vaccinations).	[180]
Reports of death	J&J/Janssen	Clinicians from the CDC and FDA examined death reports submitted to Vaccine Adverse Event Reporting System (VAERS), which may include death certificates, autopsies, and medical records. Nine deaths that can be directly linked to the J&J/Janssen COVID-19 vaccine have been found via ongoing surveillance. The CDC and FDA keep track of reports of fatalities following COVID-19 vaccinations and update data when it becomes available.	[178]
Bell’s palsy	(Nasalflu, Berna Biotech, Leiden, The Netherlands)	Bell’s palsy is a disorder that causes the muscles on one side of the face to suddenly weaken. The weakness often subsides over a few weeks and is only transitory. The weakening makes the lower portion of the face look sagging. One-sided smiles causes the afflicted eye to resist closing.	[181,182]
Postural orthostatic tachycardia syndrome (POTS)	mRNA-based vaccines	After standing or sitting up, a condition known as postural tachycardia syndrome (PoTS) causes an unnatural rise in heart rate. Consistent signs include fainting and dizziness. Postural orthostatic tachycardia syndrome (POTS) is another name for it.	[183]

**Table 4:** Difference between Viral Vector Vaccine and RNA Vaccines.

Viral Vector Vaccine	RNA Vaccine	References
Enhanced immunological reaction	Lower immunogenicity.	[202]
Ad vaccines can be kept at 2–8 °C for three–six times longer than the mRNA vaccine made by Moderna	The Moderna vaccine needs to be stored at −20 °C while the Pfizer-BioNTech vaccine needs to be kept at −70 °C. Despite the fact that each vaccine can be kept at 2–8 °C for 5 and 30 days, respectively, these rigorous long-term storage specifications will make distribution difficult, particularly in places lacking a cold-chain infrastructure.	[203]
The manufacturing process is more complicated.	In comparison to a facility handling viral particles, mRNA vaccine may offer a low-cost, cell-free production method that is highly scalable and simpler to establish and run.	[204,205]
Risk of genomic integration.	mRNAs do not pose a risk for genome integration.	[202,206]
Response dampened by pre-existing immunity against vector.	Responses to mRNA boosters are influenced by pre-existing immunity.	[207,208]
Emergency Use listing Adenoviral vector-based mostly contains Full-length Spike protein, however Ad26Cov2-S vaccine express S protein contains K986P and V987P alterations (2P) in a loop that abuts the S2’ membrane fusion machinery’s core helix.	BioNTech-Pfizer and Moderna’s mRNA vaccines have the two stabilizing mutations in S2 (K986P and V987P), which have been shown to inhibit the conformational transition of the pre-fusion into the post-fusion structure of S.	[209,210]

## Data Availability

Not applicable.
